# Frequencies of Private Mentions and Sharing of Mammography and Breast Cancer Terms on Facebook: A Pilot Study

**DOI:** 10.2196/jmir.7508

**Published:** 2017-06-09

**Authors:** Marco Huesch, Alison Chetlen, Joel Segel, Susann Schetter

**Affiliations:** ^1^ Milton S Hershey Medical Center Department of Radiology, Public Health Sciences Penn State College of Medicine Hershey, PA United States; ^2^ Milton S Hershey Medical Center Department of Radiology, Breast Imaging Division Penn State College of Medicine Hershey, PA United States; ^3^ Milton S Hershey Medical Center Department of Health Policy and Administration Pennsylvania State University University Park, PA United States; ^4^ Penn State Cancer Institute Penn State University Hershey, PA United States

**Keywords:** Facebook, online social network, social media, breast cancer screening, mammography, user comments, websites, links

## Abstract

**Background:**

The most popular social networking site in the United States is Facebook, an online forum where circles of friends create, share, and interact with each other’s content in a nonpublic way.

**Objective:**

Our objectives were to understand (1) the most commonly used terms and phrases relating to breast cancer screening, (2) the most commonly shared website links that other women interacted with, and (3) the most commonly shared website links, by age groups.

**Methods:**

We used a novel proprietary tool from Facebook to analyze all of the more than 1.7 million unique interactions (comments on stories, reshares, and emoji reactions) and stories associated with breast cancer screening keywords that were generated by more than 1.1 million unique female Facebook users over the 1 month between November 15 and December 15, 2016. We report frequency distributions of the most popular shared Web content by age group and keywords.

**Results:**

On average, each of 59,000 unique stories during the month was reshared 1.5 times, commented on nearly 8 times, and reacted to more than 20 times by other users. Posted stories were most often authored by women aged 45-54 years. Users shared, reshared, commented on, and reacted to website links predominantly to e-commerce sites (12,200/1.7 million, 36% of all the most popular links), celebrity news (n=8800, 26%), and major advocacy organizations (n=4900, 15%; almost all accounted for by the American Cancer Society breast cancer site).

**Conclusions:**

On Facebook, women shared and reacted to links to commercial and informative websites regarding breast cancer and screening. This information could inform patient outreach regarding breast cancer screening, indirectly through better understanding of key issues, and directly through understanding avenues for paid messaging to women authoring and reacting to content in this space.

## Introduction

Nearly 3 million women have a history of breast cancer today in the United States [1], and a further 15 million receive screening mammography annually [2]. Understanding how women interact with their support and social networks may be clinically important in breast cancer [3]. Breast cancer screening with imaging is widely recognized as lifesaving [4,5], yet still far too few women take advantage of this program. Widespread estimates that as many as 1 in 3 women remain unscreened or underscreened [6] suggest that more must be done to drive awareness, improve access, and increase screening.

Online social media and social networks potentially provide an opportunity for women to become aware, or more aware, of breast cancer risk and screening options and methods. Such novel channels can allow women to share intimate information regarding their symptoms, signs, screening, diagnosis, and treatment with close friends and relatives. In this study, we explored content relating to breast cancer screening on the leading US online social networking platform. Our approach has several key differentiators from past and current work.

First, we listened rather than reaching out and teaching or communicating. We sought to illustrate that researchers can use an online platform to listen to users in a way that respects their privacy and doesn’t identify them or any of their actual text. This social experience can be viewed through the lens of social normative theory, recognizing that these online channels allow users to build relationships and potentially influence the attitudes and behaviors of connected others [7,8]. The information spread through social media, whether true or false, can affect the social norms of others for good or bad, making listening to such content important for understanding perceptions, awareness, and attitudes [9].

Yet most research using Facebook, including our own, has hinged on outreach instead of listening. While an online social network is designed to be a social experience for its users, commercial outreach by advertisers and researchers is simple and cost effective. Such outreach methods exploit the personal and intimate setting afforded by the network and its highly tailored ability to finely target users based on expressed and inferred interests. For example, we reached more than 50,000 white, Latino, and Hispanic American women with an interest in maternity care in Los Angeles in part through targeted Facebook advertisements [10]. Other researchers have seen success with similar Facebook-based outreach in the settings of mental health [11,12], tobacco use [13], and drug and alcohol use [14].

Second, Facebook is an intrinsically different platform from other online platforms. Recently, Rosenkrantz and colleagues provided an innovative and important look at how women perceive the mammography experience through examination of several hundred carefully selected tweets both before and after the screening [15]. Others have similarly examined Twitter [16], YouTube video comments [17], smartphone apps [18], and Google Trends [19].

However, these other platforms differ in use, beliefs, attitudes, experiences, typical audience, and context of use. Facebook allows its users to experience gratification from satisfying the need to belong and the need for self-presentation [20,21]. Facebook also differs from the more public platforms by allowing users to share content with a circle of connected users. This offers potentially an opportunity to listen to more nuanced and private, sensitive conversations. This aspect of nonpublic, sensitive information is similar to that revealed through private searches on Google, but differs from the usually public comments on a video on YouTube, tweets on Twitter, or a weblog (blog). Other differences that distinguish Facebook are that it may, unfortunately, also allow inaccurate information, myths, or undesirable social norms to spread, unlike more public communications such as tweets, in which such issues can be more quickly and easily identified [22].

Third, the scale of our data source exceeds those of other studies leveraging Facebook data. Some studies have examined the rate of engagement with sampled posted Facebook content on breast cancer screening [23,24], or relatively small samples of conversations about complementary medicine and breast cancer on Facebook [25], or within samples of Facebook groups specifically relating to breast cancer [26].

Yet there is a wide and deep penetration of Facebook in the United States. More than half of all American adults are users [27]. It is also the most demographically representative of all online social networks; of adult women who are online, 77% are users of Facebook [28]. The Pew Research Center also found the median number of Facebook friends to be around 200 [27]. This suggests that there are large numbers of connected users who can see, comment on, and react to content that their friends create. Since such content could have positive or negative public health effects, we contend that understanding what is being shared is critical.

We believe that online investigations are crucial to understanding women’s experiences better, and to inform strategies that seek to deal with obstacles to improved utilization of screening. This pilot study is a cursory first step: an exploration of the terms and phrases used by female users on Facebook relating to breast cancer screening over a 1-month period. Our hypothesis was that adult women would be actively generating content and interacting with other users’ content on Facebook on the topic of breast cancer screening. Our objectives were to understand (1) the most commonly used terms and phrases relating to breast cancer screening, (2) the most commonly shared website links that other women interacted with, and (3) the most commonly shared website links, by age groups.

## Methods

We contracted with Sysomos Scout (Sysomos, Toronto, ON), a commercial infomediary that resells Twitter, Facebook, blog, and other social media data [10]. We provided a list of 69 keywords and key phrases (Textbox 1) to Sysomos, looking only at Facebook data generated by self-identified female users of Facebook, covering professions and organizations, formal and informal terms for services rendered relating to breast cancer and breast cancer screening, related symptoms and signs, risk strata, and investigation findings. A practicing breast imaging radiologist (AC) and a physician scientist with substantial prior experience using online infomediaries (MH) prespecified these keywords based on guidelines content, institutional patient education materials, and the bibliography of breast cancer screening literature of this study.

**Figure 1:**
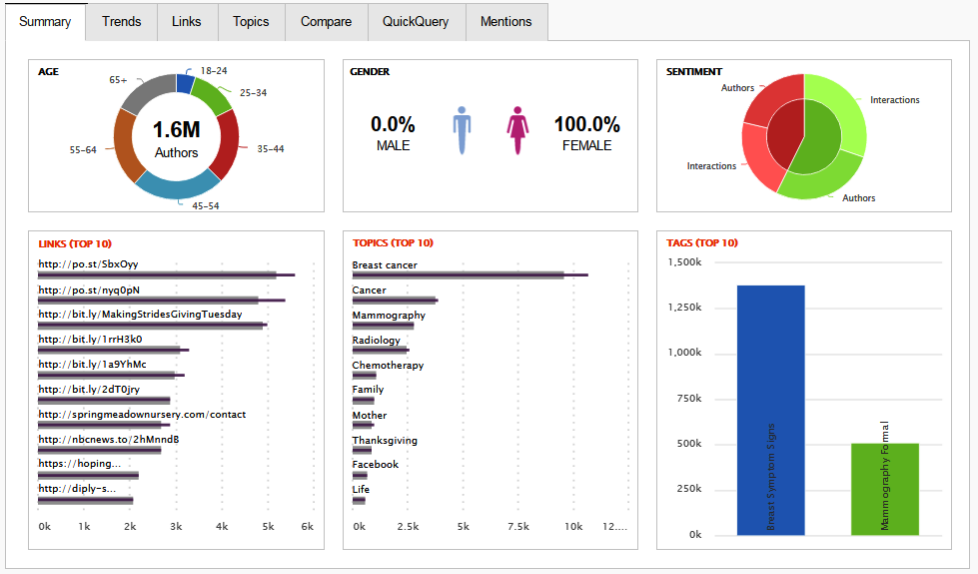
Sysomos Subscriber Dashboard screenshot showing total authors, sex and age distributions, sentiment, top links shared, and top inferred topics (source: Sysomos).

We controlled searches using the proprietary tool’s user interface (Figure 1). Facebook and Sysomos make only 30-day rolling period data available, and we randomly initiated coverage on November 15, 2016, which ran through December 15, 2016.

### Definition of Content

Sysomos matched these keywords to any Facebook *story* (a posted item of content by a user) or any type of Facebook *interaction* that can be a *reshare* (a reposting of an original story to another user connected to the sharing user), a *comment* (a text comment made by a connected user on the original story or on a prior comment), or a *reaction* (one of several emoji representing emotions, such as positive, negative, empathetic, surprise, and love).

### Definition of Counts

All counts for numbers of stories and interactions are unique, by Facebook’s construction of nonoverlapping categories of story, reshare, comment, and reaction. Counts of authors are more complex. Within a category, the number of authors is the unique number of authors. For example, if 45,000 women commented on an article, these are nonduplicated authors. Across categories this may not hold, as the same author may post several stories, comment on other stories, and react to many others.

Accordingly, we cannot add the numbers of authors across the different categories of interactions. For example, 1.1 million unique authors making reactions and the 0.4 million unique authors making comments cannot be added to obtain 1.5 million authors, because this resulting sum double counts women doing both. However, the actual total is no smaller than 1.1 million and no larger than 1.5 million. We conservatively report only the lower number and use phrases such as “…at least…” in reporting these totals.

### Most Commonly Mentioned Terms

Sysomos reported to us summary aggregate statistics such as totals, time-based trends such as subtotal by day, content-based subtotals, keyword prevalence, other word prevalence in context of keyword, and most popular website links that were posted or shared. Importantly, Facebook explicitly limits some aggregate data to just the top 10 items within a category and limits all aggregate data to items with at least 100 instances. This is due to confidentiality concerns and the ability otherwise to potentially reidentify individuals. We provide selected excerpts of these data, including tabular and graphical summaries.

### Most Commonly Shared Website Links by Interaction Type and by Age Group

In this pilot study, we were most interested in the type of content that was being shared. Links to website content originate in a story. Such stories can be authored by women who embed a link in a posted story, or authored by a marketer or news media organization that uses a shortened (eg, bitly) Web address to allow ease of use and visibility. Sysomos allowed us to identify the actual 10 most popular links and the frequency of each, by interaction type and content of link.

We clicked through all of these links and examined their content in detail. One study team member, a physician scientist (MH), manually categorized their content retroactively. This led to us identifying 5 mutually exclusive and collectively exhaustive categories to which all shared links belonged. These categories were *e-commerce related to breast cancer*, *celebrity breast cancer information*, *breast cancer advocacy and charity*, *noncelebrity breast cancer information*, and *unrelated to breast cancer*. This last category arose because, although a user may have been commenting on a breast cancer news story, they may also have been sharing an unrelated news item in the same post, and hence both were captured. These categories have not been externally validated and should be considered hypothesis generating only.

This study was conducted using completely deidentified, aggregated summary data provided by a third party, and accordingly did not involve human participant research and did not require an institutional review board determination or approval in our institution.

**Keywords and key phrases used to capture Facebook data.**

**Profession and organizational terms**

Radiology

Doctor xray

Hospital Xray

Hospital Radiology

Radiologist

breast center

breast imaging center

breast cancer screening

breast screen guidelines

breast screening guidelines

**Services rendered: formal terms**

Mammogram

Mammography

Breast Exam

Digital mammography

digitized mammographic image

Breast tomosynthesis

three-dimensional mammography

three-dimensional mammogram

3-D mammogram

3d mammogram

3d mammogram

breast imaging

breast image

full-field digital mammogram

Screening Mammography

Screening Mammogram

Diagnostic Mammography

Diagnostic Mammogram

3-D mammography

mastectomy

3d mammography

Lumpectomy

full-field digital mammography

digital breast tomosynthesis

3d mammography

breast tumor

Digital mammogram

breast needle biopsy

**Services rendered: informal terms**

breast xray

breast x-ray

Xray of my breasts

X-ray of my breasts

X-ray of my breast

Doctor x-rayed my breasts

Hospital X-rayed my breasts

x-rayed my breasts

needle biopsy done of my breast

needle biopsy of my breast

**Symptoms and signs terms**

breast lump

breast lumps

lump in my breast

**Preexisting risk terms**

BRCA tested positive

BRCA positive

family risk breast

high-risk breast

high-risk breasts

**Findings terms**

abnormal breast screen

abnormal breast x-ray

abnormal breast xray

dense breast

dense breasts

breast density

DCIS

ductal carcinoma

fatty breasts

fatty breast

breast cancer

## Results

More than 1.7 million unique interactions (comments on stories, reshares, and emoji reactions) and stories associated with the 69 breast cancer screening keywords were generated by at least 1.1 million Facebook users over the 30-day period from November 16, 2016 to December 15, 2016.

On average, each of the 59,000 unique stories during the month was reshared 1.5 times, commented on nearly 8 times, and reacted to more than 20 times by other users seeing the original content.

### Most Commonly Mentioned Terms

**Figure 2:**
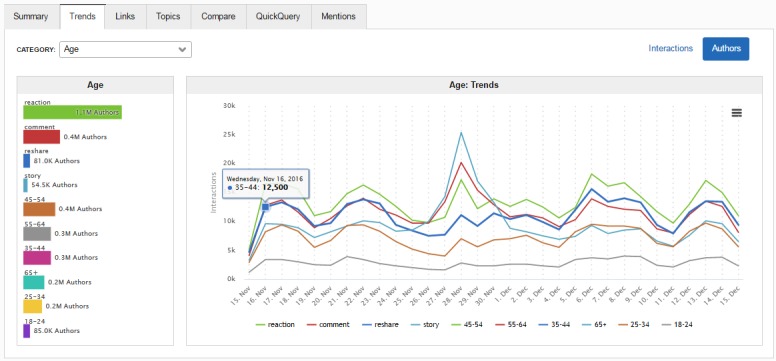
Sysomos Subscriber Dashboard screenshot showing trends in interaction types and age groups daily over the 30-day rolling time period (November 15-December 15, 2016) (source: Sysomos).

Stories and interactions were most often authored by women aged 45-54 years (Figure 2). We observed a substantial spike in volume on November 28, 2016, the Monday on which many news sites shared a picture of a bald Ms Shannen Doherty (an American actress) and her mother, immediately prior to Ms Doherty’s radiotherapy.

A search of mentions of “Doherty” in any link shared, reshared, or otherwise interacted with showed 6700 mentions by 6600 unique authors over the month, respectively 0.4% and 0.6% of the overall totals for the month.

Common terms relating to breast cancer and screening mammography mentioned in any context included “mammogram” (266,000 interactions, or 16% of the month’s total interactions), “lump” (26,600, 1.6%), “abnormal mammogram” (4400, 0.3%), “scars” (4000, 0.2%), “BRCA” (3800, 0.2%), “dense” (3200, 0.2%), “DCIS” (3000, 0.2%), “high risk” (2900, 0.2%), and “compression” (1000, 0.06%).

### Most Commonly Shared Website Links by Interacted Type

Across all interactions, the 10 most popular links accounted for a total of 33,600 interactions, or almost 2% of monthly total interactions (Table 1). Multimedia Appendix 1 provides the actual links.

Links to *e-commerce related to breast cancer* represented the plurality of interacted links at 36% of all top interactions and were the most popular to be reshared with connected users (59% of all top reshares). These tended to represent a for-profit or not-for-profit organization that was selling items in some way connected to breast cancer themes over the Web. The most common of these were thebreastcancersite.com, hopinghand.com, and makaiclothing.com. Other links in this category were also aimed at recruiting email addresses for future direct marketing by offering giveaways (thebreastcancersite.com).

The next largest category was links to *celebrity breast cancer information*, which represented 26% of all top interactions and originated in stories such as from the television program *The Today Show*, E! Online, and similar sites. Here the most prominent foci were Ms Shannen Doherty, Ms Danielle Spencer, and Ms Robin Roberts, in that order. Almost all of these interactions were emoji-based reactions.

The third largest category represented links to *breast cancer advocacy and charity* sites, with 15% of all top interactions concentrated among links to 2 sites. The American Cancer Society donation page (“Making Strides Giving Tuesday”) was the second most popular link interacted with overall, with 4900 interactions or 0.3% of all interactions, was the most reacted to, and was the fifth most often reshared link among all top links. The other site was the advocacy and information site of Susan G. Komen, which did not make the top 10 links overall, and represented only 100 commented-on links (the 10th most commented-on link).

*Noncelebrity breast cancer information* links constituted only 6% of all top interactions and included mostly personal blog stories, inspirational messages, and some traditional news media. The most prominent themes in this category were positive stories around the use of tattoos to mask the surgical scars associated with breast cancer surgery and a new breast cancer vaccine trial at City of Hope hospitals in Duarte, California.

Importantly, in this category were at least 700 shared links relating to mercola.com, a natural health advocacy site that presented a view against breast cancer screening, including multiple references to scientific studies and a recent article by Welch and colleagues [29]. This single link alone represented nearly 6% of all the top links in the 35- to 54-year age group.

Finally, more than 1 in 6 links were not in relation to breast cancer or screening terms. These presumably were stories, reshares, and comments in which a user conveyed multiple messages, some about breast cancer (hence they were selected by Sysomos) and some not about this.

### Most Commonly Shared Website Links by Age Group

We repeated our analyses to understand how interest and interactions changed across age groups (Table 2). Examining the totals of the top 10 links by age group, we found a clear increase with age. The most salient findings were the increase by age groups in e-commerce, peaking in the 45- to 54-year age group, and the complete or nearly complete lack of interest in those between 18 and 44 years of age in breast cancer advocacy and charity sites.

Additionally, we noted that noncelebrity-related news and information about breast cancer represented a larger share among the youngest users (50%) than among older users. We also noted the apparent complete lack of interest among the age group 65 years and older in celebrity-related breast cancer information.

**Table 1:** Distribution of most popular links by category and interaction type.

Top 10 links	Most popular reshared with others	Most commented on	Most reacted to	Overall most popular across all interactions
E-Commerce related to breast cancer	3100 (59%)	400 (29%)	9400 (32%)	12,200 (36%)
Celebrity breast cancer information	300 (6%)	500 (36%)	8000 (28%)	8800 (26%)
Noncelebrity breast cancer information	1000 (19%)	300 (21%)	1700 (6%)	2100 (6%)
Breast cancer advocacy and charity	300 (6%)	100 (7%)	4500 (16%)	4900 (15%)
Unrelated content	600 (11%)	100 (7%)	5400 (19%)	5600 (17%)
Total of top 10 link volume	5300	1400	29,000	33,600

**Table 2:** Distribution of most popular links by category and age group.

Top 10 links	Age group (years)						Overall most popular across all interactions
	18-24	25-34	35-44	45-54	55-64	≥65	
E-Commerce related to breast cancer	100 (7%)	900 (28%)	2200 (37%)	3500 (59%)	3400 (40%)	3100 (35%)	12,200 (36%)
Celebrity breast cancer information	200 (14%)	900 (28%)	2200 (37%)	2500 (42%)	1100 (13%)	0 (0%)	8800 (26%)
Noncelebrity breast cancer information	700 (50%)	200 (6%)	600 (10%)	400 (7%)	900 (11%)	1600 (18%)	2100 (6%)
Breast cancer advocacy and charity	0 (0%)	0 (0%)	300 (5%)	600 (10%)	1500 (17%)	3100 (35%)	4900 (15%)
Unrelated content	0 (0%)	400 (13%)	700 (12%)	900 (15%)	1700 (20%)	1100 (12%)	5600 (17%)
Total of top 10 link volume	1400	3200	6000	5900	8600	8900	33,600

## Discussion

In this novel pilot study, we examined aggregated mentions of terms and phrases, and shared website links among women in the United States on Facebook in relation to breast cancer screening over a 1-month window. We found substantial content posted by, shared among, and interacted with by large numbers of women. The most popular stories provided information on women undergoing treatment for breast cancer and information on online destinations to purchase small items and make small donations to further research.

We observed that the timing of upswings in interest often appeared to coincide with celebrity news, such as a picture shared by Shannen Doherty of herself about to receive radiotherapy for her breast cancer. In general, our work supports the importance of sharing of and commenting on stories about well-known celebrities with breast cancer [30,31].

It is well-known that the Internet allows a so-called long tail to form, in which many niche sites, topics, or products are, respectively, visited, mentioned, or bought by a small number of users, in contrast with more popular sites, topics, or products [32]. We found a limited count of women creating and interacting with very popular content, such as content relating to “mammogram” (more than 15% of all interactions in our study). For example, we found that even many of the most popular terms, such as “DCIS” and “dense,” represented very small (0.2%) proportions of the overall number of story interactions. These may nevertheless be a meaningful subgroup. We saw the same phenomenon with interactions to links. The 10 most popular links accounted for just 2% of all interactions. This suggests that many items of less popular content were still, in aggregate, accounting for a large number of shared links.

Yet, despite these restrictions, we found that there was a plurality of links to commercial e-commerce websites marketing items related to breast cancer themes, such as thebreastcancersite.com. We saw little sharing of original medical news content from formal online media or formal health information publishers, despite the positive impact this can have [33]. There was some sharing of a story from a natural health website that appeared to be strongly against breast cancer screening. The extent of sharing of this site (nearly 6% of all the top links in the 35- to 54-year age group) and the strength of the views against breast cancer screening in the content on that site appeared to us to echo well-known online campaigns against vaccines by vaccine skeptics [34].

We also found less content than we had expected from some of the most prominent advocacy organizations, such as Susan G. Komen, although the American Cancer Society’s breast cancer site was the link with the second most frequent interactions. Finally, we saw fewer mentions than we had expected of terms anecdotally thought to be points of concern for women (eg, breast compression during imaging) and that had been found among their tweets in a recent innovative study by Rosenkrantz and colleagues [15]. One potential limitation is that, given the terms of use of the tool, we were able to examine only a 1-month study window.

As we continue to examine this new data source, we expect to obtain more detailed insights about what women are interacting about and how they are interacting regarding breast cancer terms. We expect that such data can inform the outreach of advocacy organizations, and can inform campaigns to improve rates of screening and to educate high-risk women concerning their options, among many other examples.

Methodologically, this study adds to our understanding of patients’ and consumers’ articulated thoughts and feelings about important public health initiatives such as breast cancer screening. We showed that summarized information is available from the world’s leading online social network, and note that this commercially available information is distinct from more easily analyzed public online social media. Given the greater demographic representativeness of Facebook, compared with other online social media and social networks [8], the data on this platform are a potentially useful research tool.

### Limitations

While our study had several important strengths, including novelty, exhaustiveness, and national scale in the United States, there are several important limitations. Our data source, Sysomos, is a commercial reseller of data obtained indirectly from Facebook through another intermediary, Datasift. Data provenance, custody, and governance must be assumed but cannot be verified or guaranteed. For example, software errors could occur at each one of these handoffs, as well as within each segment of the data custody chain.

In particular, Facebook is the data owner, whose terms of service do not permit actual visualization of the original post or comment. To protect users’ privacy, all data were aggregated, deidentified, and mapped coarsely into topics. We therefore had no independent ability to confirm whether the reported statistics we obtained were accurate, representative, or exhaustive. Moreover, under our contract, data availability was limited to rolling 1-month lookback periods. Other restrictions motivated by privacy and imposed by the data owner include sampling only high-frequency items, limiting results to the top 10 items in a category, and masking results in which fewer than 100 Facebook users mentioned a term or shared a link. As a result, none of our results were able to provide a full view of the frequency distribution.

Neither we nor other researchers can subsequently return to historical periods beyond examining reports that were downloaded contemporaneously. Similarly, only a 30-day rolling period of aggregated data is made available by Facebook, Datasift, and Sysomos. This clearly further limits replication and error checking. For research purposes, while substantial information abides, much is lost during this process. This weakness does not seem to be one that will be alleviated, given legitimate concerns regarding online privacy [35].

Finally, while our research is internally valid, the extent to which it is externally applicable is not known. The particular month of data we looked at was almost immediately after a polarizing general election in the United States, in which health-related conversations (eg, Affordable Care Act, Planned Parenthood, women’s right to choose) were widely occurring. In other months, there might have been fewer mentions of breast cancer screening terms. Our research also explicitly required women to have access to the Internet, be a member of Facebook, and use English in their interactions. There are clearly large parts of US society in which one or more of these requirements are not met.

Future researchers may exploit other less coarse methods for obtaining online social media and social network data. Companies operating online survey panels such as Knowledge Networks, Inc [36], Qualtrics, and ClearVoice Research [37] can allow more representative surveys and more specific questions as to what women share on Facebook. Free resources can also be accessed through Google’s own Trends data—for example, to analyze searches related to Angelina Jolie’s disclosed prophylactic mastectomies [8], and to understand interest in public hospital quality reports [38]—or by exploiting the freely available Twitter data [39].

### Conclusions

Examining novel data from the universe of mentions on the leading online social network regarding breast cancer screening-related terms provided an important but superficial and initial look at topics of great interest among all female Facebook users over 1 month. More work is needed using this novel data source and applying its insights to solving pressing public health problems, including the inadequate screening for breast cancer.

## Appendix

Multimedia Appendix 1 Categorization of top 10 links by interaction type and by age category.

## References

[1] (2016). U.S. breast cancer statistics.

[2] National Center for Health Statistics (2017). Mammography.

[3] Kroenke CH, Kubzansky LD, Schernhammer ES, Holmes MD, Kawachi I (2006). Social networks, social support, and survival after breast cancer diagnosis. J Clin Oncol.

[4] Chetlen A, Mack J, Chan T (2016). Breast cancer screening controversies: who, when, why, and how?. Clin Imaging.

[5] Webb ML, Cady B, Michaelson JS, Bush DM, Calvillo KZ, Kopans DB, Smith BL (2014). A failure analysis of invasive breast cancer: most deaths from disease occur in women not regularly screened. Cancer.

[6] U.S. Department of Health and Human Services, Centers for Disease Control and Prevention, National Center for Health Statistics (2016). Health, United States, 2015: with special feature on racial and ethnic health disparities.

[7] Hanson CL, Cannon B, Burton S, Giraud-Carrier C (2013). An exploration of social circles and prescription drug abuse through Twitter. J Med Internet Res.

[8] Huesch M (2015). Celebrity influence? An observational study of consumer responses on social media and on an internet search engine to news of Angelina Jolie?s prophylactic mastectomies. CESR-Schaeffer Working Paper Series, Paper No-2015-012.

[9] Hanson CL, Burton SH, Giraud-Carrier C, West JH, Barnes MD, Hansen B (2013). Tweaking and tweeting: exploring Twitter for nonmedical use of a psychostimulant drug (Adderall) among college students. J Med Internet Res.

[10] Huesch MD, Galstyan A, Ong MK, Doctor JN (2016). Using social media, online social networks, and internet search as platforms for public health interventions: a pilot study. Health Serv Res.

[11] Batterham PJ (2014). Recruitment of mental health survey participants using Internet advertising: content, characteristics and cost effectiveness. Int J Methods Psychiatr Res.

[12] Pedersen ER, Helmuth ED, Marshall GN, Schell TL, PunKay M, Kurz J (2015). Using facebook to recruit young adult veterans: online mental health research. JMIR Res Protoc.

[13] Carter-Harris L, Bartlett ER, Warrick A, Rawl S (2016). Beyond traditional newspaper advertisement: leveraging Facebook-targeted advertisement to recruit long-term smokers for research. J Med Internet Res.

[14] Fazzino TL, Rose GL, Pollack SM, Helzer JE (2015). Recruiting U.S. and Canadian college students via social media for participation in a web-based brief intervention study. J Stud Alcohol Drugs.

[15] Rosenkrantz AB, Labib A, Pysarenko K, Prabhu V (2016). What do patients tweet about their mammography experience?. Acad Radiol.

[16] Thackeray R, Burton SH, Giraud-Carrier C, Rollins S, Draper CR (2013). Using Twitter for breast cancer prevention: an analysis of breast cancer awareness month. BMC Cancer.

[17] Basch CH, Hillyer GC, MacDonald ZL, Reeves R, Basch CE (2015). Characteristics of YouTube™ videos related to mammography. J Cancer Educ.

[18] Coughlin SS (2014). Intervention approaches for addressing breast cancer disparities among African American women. Ann Transl Med Epidemiol.

[19] Fazeli DS, Carlos RC, Hall KS, Dalton VK (2014). Novel data sources for women's health research: mapping breast screening online information seeking through Google Trends. Acad Radiol.

[20] Joison A (2008). 'Looking at', 'looking up' or 'keeping up with' people? Motives and uses of Facebook.

[21] Nadkarni A, Hofmann SG (2012). Why do people use Facebook?. Pers Individ Dif.

[22] Gabarron E, Serrano JA, Wynn R, Lau AY (2014). Tweet content related to sexually transmitted diseases: no joking matter. J Med Internet Res.

[23] Hale TM, Pathipati AS, Zan S, Jethwani K (2014). Representation of health conditions on Facebook: content analysis and evaluation of user engagement. J Med Internet Res.

[24] Theiss SK, Burke RM, Cory JL, Fairley TL (2016). Getting beyond impressions: an evaluation of engagement with breast cancer-related Facebook content. Mhealth.

[25] Mazzocut M, Truccolo I, Antonini M, Rinaldi F, Omero P, Ferrarin E, De PP, Tasso C (2016). Web conversations about complementary and alternative medicines and cancer: content and sentiment analysis. J Med Internet Res.

[26] Bender JL, Jimenez-Marroquin M, Jadad AR (2011). Seeking support on facebook: a content analysis of breast cancer groups. J Med Internet Res.

[27] Smith A (2014). 6 new facts about Facebook.

[28] Duggan M (2015). The demographics of social media users.

[29] Welch HG, Prorok PC, O'Malley AJ, Kramer BS (2016). Breast-cancer tumor size, overdiagnosis, and mammography screening effectiveness. N Engl J Med.

[30] Juthe RH, Zaharchuk A, Wang C (2015). Celebrity disclosures and information seeking: the case of Angelina Jolie. Genet Med.

[31] (2014). PressRun: The New York Times's most visited content of 2013.

[32] Karch M (2016). What is the long tail and how does it apply to Google?.

[33] Price J, Simon K (2009). Patient education and the impact of new medical research. J Health Econ.

[34] Abbasi A, Adjeroh D, Dredze M, Paul MJ, Zahedi FM, Zhao H, Walia N, Jain H, Sanvanson P, Shaker R, Huesch MD, Beal R, Zheng W, Abate M, Ross A (2014). Social media analytics for smart health. IEEE Intell Syst.

[35] Huesch MD (2014). Privacy protection during internet search for health-related information--reply. JAMA Intern Med.

[36] Doctor JN, Huesch MD, Meeker D (2016). Rethinking the value of survival: clinical trials should measure patient preferences for survival on entry to trials. J Clin Epidemiol.

[37] Huesch M, Brady R (2015). Should past recipients of a kidney be allowed to have another? Surveys of preferences of individuals with and without end stage renal disease. USC CESR-Schaeffer Working Paper Series, Paper No-2015-006.

[38] Huesch MD, Currid-Halkett E, Doctor JN (2014). Public hospital quality report awareness: evidence from national and Californian Internet searches and social media mentions, 2012. BMJ Open.

[39] Huesch M, Ver Steeg G, Galstyan A (2013). Vaccination (anti-) campaigns in social media. https://www.aaai.org/ocs/index.php/WS/AAAIW13/paper/view/7094/6502.

