# Supplementary material for: Frequencies of Private Mentions and Sharing of Mammography and Breast Cancer Terms on Facebook: A Pilot Study
Source: J Med Internet Res. 2017 Jun 9;19(6):e201. doi: 10.2196/jmir.7508 (PMC5482928; doi:10.2196/jmir.7508)
Supplement: Multimedia Appendix 1 [file jmir_v19i6e201_app1.pdf]

Appendix 1: Categorization of top 10 links by interaction type

33,600 most popular links overall (of all interactions by 1,681,400 authors)
http://po.st/SbxOyy TheBreastCancerSite.com giveaway
5,200
http://bit.ly/MakingStridesGivingTuesday American Cancer Society donation page

4,900
http://po.st/nyq0pN TheBreastCancerSite.com giveaway
4,800
http://bit.ly/1rrH3k0 BlackDoctor.org info on Danielle Spencer breast cancer
3,100
http://bit.ly/1a9YhMc BlackDoctor.org info on Robin Roberts breast cancer

3,000
http://bit.ly/2dT0jry Unrelated product advertisement
2,900
http://springmeadownursery.com/contact Unrelated service announcement
2,700
http://nbcnews.to/2hMnndB Today.com Shannen Doherty breast cancer
2,700
https://hopinghand.com/collections/frontpage/products/cancer-necklace E-commerce site

2,200
http://diply-smile.com/auntyacid/article/tattoos-inspirational-art Tattoos to hide surgical scars
2,100

5,300 most reshared links (of all reshares by 81,000 authors)
http://po.st/SbxOyy TheBreastCancerSite.com giveaway
1,500
http://po.st/nyq0pN TheBreastCancerSite.com giveaway
1,300
http://articles.mercola.com/sites/articles/archive/2014/02/26/mammograms.aspx Anti-screening
500
http://lernervilletickets.com/products/monsters-at-lernerville Unrelated service announcement
400
http://bit.ly/MakingStridesGivingTuesday American Cancer Society donation page
300
http://trib.al/icgMyh0 Tattoos to hide surgical scars
300

https://hopinghand.com/collections/frontpage/products/cancer-necklace E-commerce site

300
http://bit.ly/1rrH3k0 BlackDoctor.org info on Danielle Spencer breast cancer
300
http://diply-smile.com/auntyacid/article/tattoos-inspirational-art Tattoos to hide surgical scars 200
http://fox5ny.com/news/[REDACTED]89-story Not classifiable
200

1,400 most commented links (of all comments by 420,600 authors)

http://littlethings.com/shannen-doherty-first-radiation-treatment Online media Shannen Doherty
200
http://nbcnews.to/2hMnndB Today.com Shannen Doherty breast cancer
200
http://po.st/3yJ9E4 Commercial e-commerce site with breast-cancer related items
200
http://po.st/SbxOyy TheBreastCancerSite.com giveaway
200
http://caringbridge.org/visit/daniellebyington Online social network for health problems
200
http://bit.ly/1a9YhMc BlackDoctor.org info on Robin Roberts breast cancer
100
http://plaindealer-sun.com/main.asp?ArticleID=47409&SectionID=24&SubSectionID=85&TM=50375.36 Unrelated news item

100
http://brookeaturner.com/2016/12/another-divine-adventure Personal blog re breast cancer
100
http://sgk.mn/2eOSFUh Advocacy and informational site of Susan G. Komen
100

29,000 most reacted to links (of all interactions with links by 1,125,300 authors)

http://bit.ly/MakingStridesGivingTuesday American Cancer Society donation page
4,500
http://po.st/SbxOyy TheBreastCancerSite.com giveaway
3,800
http://po.st/nyq0pN TheBreastCancerSite.com giveaway
3,700
http://bit.ly/1rrH3k0 BlackDoctor.org info on Danielle Spencer breast cancer
2,800

http://bit.ly/1a9YhMc BlackDoctor.org info on Robin Roberts breast cancer
2,800
http://bit.ly/2dT0jry Unrelated product advertisement
2,800
http://springmeadownursery.com/contact Unrelated service announcement
2,600
http://nbcnews.to/2hMnndB Today.com Shannen Doherty breast cancer
2,400
https://hopinghand.com/collections/frontpage/products/cancer-necklace E-commerce site

1,900
http://diply-smile.com/auntyacid/article/tattoos-inspirational-art Tattoos to hide surgical scars 1,700

Appendix 2: Categorization of top 10 links by age category

1,400 most popular links shared by __18-24 year olds__ (of all interactions by 85,000 authors)

http://trib.al/icgMyh0 Online media article about tattoos to hide breast cancer surgical scars

500

http://stampedeproducts.com/Products/Sidewind-Deflectors.asp Unrelated product advert

400

http://eonli.ne/2gpvXiL E-online article on Shannen Doherty

100

http://peoplem.ag/1IBC9NG Online article on TSA pat down of women with breast cancer

100

http://po.st/nyq0pN TheBreastCancerSite.com giveaway

100

http://nbcnews.to/2hMnndB Today.com Shannen Doherty breast cancer

100

http://abcn.ws/2fcPPab News article on teacher with breast cancer

100

3,200 most popular links shared by __25-34 year olds__ (of all interactions by 216,400 authors)

http://bit.ly/2dT0jry Unrelated product advertisement

400

http://nbcnews.to/2hMnndB Today.com Shannen Doherty breast cancer

400

http://stampedeproducts.com/Products/Sidewind-Deflectors.asp Unrelated product advert

400

http://makaiclothingco.com/products/limited-edition-pink-makai-brest-cancer-awareness-shirt?variant=%5BREDACTED Unclassifiable

400

http://bit.ly/1rrH3k0 BlackDoctor.org info on Danielle Spencer breast cancer

400

http://po.st/nyq0pN TheBreastCancerSite.com giveaway

300

http://bit.ly/1a9YhMc BlackDoctor.org info on Robin Roberts breast cancer

300

http://eonli.ne/2gpvXiL E-online article on Shannen Doherty

200

http://po.st/SbxOyy TheBreastCancerSite.com giveaway

200

http://trib.al/icgMyh0 Tattoos to hide surgical scars
200

6,000 most popular links shared by 35-44 year olds (of all interactions by 322,500 authors)

https://hopinghand.com/collections/frontpage/products/cancer-necklace E-commerce site

1,000
http://bit.ly/1rrH3k0 BlackDoctor.org info on Danielle Spencer breast cancer
800
http://bit.ly/1a9YhMc BlackDoctor.org info on Robin Roberts breast cancer
800
http://po.st/nyq0pN TheBreastCancerSite.com giveaway
700
http://bit.ly/2dT0jry Unrelated product advertisement
700
http://nbcnews.to/2hMnndB Today.com Shannen Doherty breast cancer
600
http://po.st/SbxOyy TheBreastCancerSite.com giveaway
500
http://sgk.mn/2eOSFUh Advocacy and informational site of Susan G. Komen
300
http://diply-smile.com/auntyacid/article/tattoos-inspirational-art Tattoos to hide surgical scars 300
http://articles.mercola.com/sites/articles/archive/2014/02/26/mammograms.aspx Anti-screening site 300

5,900 most popular links shared by 45-54 year olds (of all interactions by 369,400 authors)

http://po.st/nyq0pN TheBreastCancerSite.com giveaway
1,200
http://po.st/SbxOyy TheBreastCancerSite.com giveaway
1,100
http://bit.ly/1a9YhMc BlackDoctor.org info on Robin Roberts breast cancer
1,000
http://bit.ly/2dT0jry Unrelated product advertisement
900
http://bit.ly/1rrH3k0 BlackDoctor.org info on Danielle Spencer breast cancer
900
https://hopinghand.com/collections/frontpage/products/cancer-necklace E-commerce related to breast cancer 800

http://bit.ly/MakingStridesGivingTuesday American Cancer Society donation page
600
http://nbcnews.to/2hMnndB Today.com Shannen Doherty breast cancer
600
http://po.st/3yJ9E4 Commercial e-commerce site with breast-cancer related clothes
400
http://articles.mercola.com/sites/articles/archive/2014/02/26/mammograms.aspx Anti-screening site 400

8,600 most popular links shared by <u>55-64 year olds</u> (of all interactions by 320,400 authors)
http://po.st/SbxOyy TheBreastCancerSite.com giveaway
1,500
http://bit.ly/MakingStridesGivingTuesday American Cancer Society donation page
1,500
http://po.st/nyq0pN TheBreastCancerSite.com giveaway
1,400
http://springmeadownursery.com/contact Unrelated service announcement
1,000
http://bit.ly/2dT0jry Unrelated product advertisement
700
http://bit.ly/1rrH3k0 BlackDoctor.org info on Danielle Spencer breast cancer
600
http://bit.ly/1a9YhMc BlackDoctor.org info on Robin Roberts breast cancer
500
http://po.st/3yJ9E4 Commercial e-commerce site with breast-cancer related clothes
500
http://diply-smile.com/auntyacid/article/tattoos-inspirational-art Tattoos to hide surgical scars 500
http://social.coh.org/sL2E City of Hope hospital article on breast cancer vaccine
400

8,900 most popular links shared by <u>65+ year olds</u> (of all interactions by 242,600 authors)
http://bit.ly/MakingStridesGivingTuesday American Cancer Society donation page
2,500
http://po.st/SbxOyy TheBreastCancerSite.com giveaway
1,400
http://springmeadownursery.com/contact Unrelated service announcement
1,100
http://po.st/nyq0pN TheBreastCancerSite.com giveaway
1,000
http://social.coh.org/sL2E City of Hope hospital article on breast cancer vaccine promise
800

http://bit.ly/2fWxaNV Homepage of ACS Making Strides against Breast Cancer 600

http://po.st/8X5ugd Commercial e-commerce site with breast-cancer related items 400

http://sosharethis.com/hes-bullied-wearing-pink-school-next-day-sees-teacher-mouth-drops News article related to child with breast cancer awareness shirt 400

http://diply-smile.com/auntyacid/article/tattoos-inspirational-art Tattoos to hide surgical scars 400

http://po.st/3yJ9E4 Commercial e-commerce site with breast-cancer related clothes 300

===
